# Editorial for the Special Issue on “Feature Papers in Section AI in Imaging”

**DOI:** 10.3390/jimaging10090214

**Published:** 2024-08-31

**Authors:** Antonio Fernández-Caballero

**Affiliations:** 1 Instituto de Investigación en Informática de Albacete, 02071 Albacete, Spain; antonio.fdez@uclm.es; Tel.: +34-926-052-959; 2 Departamento de Sistemas Informáticos, Universidad de Castilla-La Mancha, 02071 Albacete, Spain

## 1. Introduction

Artificial intelligence (AI) techniques are being used by the imaging academia and industry to solve a wide range of previously intractable problems. Image recognition and understanding are considered important sub-fields of AI. In addition, topics that are at the core of AI, such as machine learning, knowledge engineering, reasoning and inference, are common to imaging researchers. Therefore, this Special Issue has provided a forum for the publication of articles describing the use of classical and modern AI methods in imaging applications (https://www.mdpi.com/journal/jimaging/special_issues/90IUESYOPP, accessed on 26 August 2024).

This Special Issue aimed to provide a collection of high-quality research articles that address broad challenges on both the theoretical and application aspects of AI in imaging. We invited colleagues to contribute original research articles as well as review articles that would stimulate the continuing effort on the application of AI approaches to solve imaging problems.

The topics of this Special Issue on “Feature Papers in Section AI in Imaging” explicitly included (but were not limited to) the following aspects: machine learning in imaging; expert systems in imaging; knowledge engineering in imaging; neural networks in imaging; intelligent agents and multi-agent systems in imaging; evolutionary and fuzzy computation in imaging; reasoning and inference in imaging; and applications of artificial intelligence in imaging.

## 2. The Published Articles

Five papers were published in this Special Issue on “Feature Papers in Section AI in Imaging”. Despite the many existing approaches in the field, this scientific area still motivates many researchers and many challenges remain. Of the five papers, four were original research papers and the last one was a systematic review article.

The first paper “Fully Self-Supervised Out-of-Domain Few-Shot Learning with Masked Autoencoders” by Walsh, Osman, Abdelaziz, and Shehata (contribution 1) addresses the problem of few-shot learning. Few-shot learning aims to identify unseen classes with limited labeled data. The most problematic aspects of few-shot learning techniques are introduced by the authors. Their paper proposes a fully self-supervised few-shot learning (FSS) technique that uses a vision transformer and a masked autoencoder. The proposed technique can generalize to out-of-domain classes by fine-tuning the model in a fully self-supervised method for each episode. The proposed technique is evaluated using the ISIC, EuroSat, and BCCD datasets (all out-of-domain). As such, the results show that FSS has an accuracy gain on the three datasets without the use of supervised training.

In the second paper, “Constraints on Optimizing Encoder-Only Transformers for Modelling Sign Language with Human Pose Estimation Keypoint Data”, Woods and Rana (contribution 2) state that understanding the effect of each hyperparameter and regularization technique on the performance of a given supervised deep learning model is paramount to research. The authors present a comprehensive, large-scale ablation study for an encoder-only transformer to model sign language, using the enhanced word-level American Sign Language (WLASL-alt) dataset and human pose estimation keypoint data, in order to constrain the potential for optimizing the task. They also measure the impact of a number of model parameter regularization and data augmentation techniques on sign classification accuracy. The article demonstrates that the model architecture is constrained by the small dataset size for this task by finding an appropriate set of model parameter regularization and common or basic dataset augmentation techniques. Furthermore, using the base model configuration, a new maximum top-1 classification accuracy of 84% on 100 signs is reported, thereby improving the previous benchmark result for this model architecture and dataset.

The third article “Threshold-Based BRISQUE-Assisted Deep Learning for Enhancing Crack Detection in Concrete Structures” by Pennada, Perry, McAlorum, Dow, and Dobie (contribution 3) addresses automated visual inspection for crack detection on the surfaces of concrete structures. However, the authors note that poor image quality significantly affects the classification performance of convolutional neural networks. Therefore, they evaluate the suitability of image datasets used in deep learning models, such as Visual Geometry Group 16 (VGG16), for accurate crack detection. Their study examines the sensitivity of the BRISQUE method to different types of image degradation, such as Gaussian noise and Gaussian blur. By evaluating the performance of the VGG16 model on these degraded datasets with varying levels of noise and blur, a correlation between image degradation and BRISQUE scores is established. The results show that images with lower BRISQUE scores achieve higher accuracy, F1 score, and Matthew’s correlation coefficient in crack classification. This study suggests the implementation of a BRISQUE score threshold to optimize training and testing times, resulting in reduced computational costs. These results have significant implications for improving the accuracy and reliability of automated visual inspection systems for crack detection and structural health monitoring.

In the fourth paper, “Data-Weighted Multivariate Generalized Gaussian Mixture Model: Application to Point Cloud Robust Registration” by Ge, Najar, and Bouguila (contribution 4), a weighted multivariate generalized Gaussian mixture model combined with stochastic optimization is proposed for point cloud registration. The mixture model parameters of the target scene and the scene to be registered are iteratively updated by the fixed-point method under the expectation–maximization (EM) algorithm, and the number of components is determined based on the minimum message length criterion. The Kullback–Leibler divergence between these two mixture models is used as the loss function for stochastic optimization to find the optimal parameters of the transformation model. The self-built point clouds are used to evaluate the performance of the proposed algorithm on rigid registration. Experiments show that the algorithm dramatically reduces the effects of noise and outliers, and effectively extracts the key features of the data-intensive regions.

The final paper by Cumbajin, Rodrigues, Costa, Miragaia, Frazão, Costa, Fernández-Caballero, Carneiro, Buruberri, and Pereira entitled “A Systematic Review on Deep Learning with CNNs Applied to Surface Defect Detection” (contribution 5) introduces a review on surface defect detection with deep learning. In this systematic review, the authors present a classification for surface defect detection based on convolutional neural networks (CNNs) focusing on surface types. The review mainly focuses on finding a classification for the most commonly used surface types in industry (metal, building, ceramic, wood, and special). Furthermore, a new machine learning taxonomy is proposed based on the results obtained and the information collected. We summarized the studies and extracted the main characteristics such as surface type, problem type, timeline, network type, techniques, and datasets. The paper shows that transfer learning was used in 83.05% of the studies, while data augmentation was used in 59.32%. The results also provide insights into the most commonly used cameras, the strategies used to overcome lighting challenges, and the approach to creating datasets for real-world applications. The key findings presented in this review allow for a quick and efficient search of information for researchers and professionals interested in improving the outcomes of their defect detection projects.

## 3. Conclusions

These five papers received a total of about 10,500 views and 20 citations, which shows the interest in this Special Issue and the scientific dynamism of the field.

According to the Top 10 Imaging Technology Trends in 2024 (https://www.startus-insights.com/innovators-guide/imaging-technology-trends/, accessed on 26 August 2024), several of these trends are directly and/or indirectly related to AI in imaging. For example, deep learning techniques that improve image classification, object detection, and segmentation are AI hot topics in imaging. It is well known that deep learning enables better identification and localization of objects in images for various applications. In our Special Issue, deep learning is at the core of applications as diverse as visual inspection, earth observation, human pose estimation, and crack detection. With its improved image classification, computer vision with deep learning is expected to find further use in many other application domains, with special attention being paid to medical imaging [1].

In fact, the latest advances in AI are transforming diagnostic imaging, and improving patient care with faster, more accurate diagnoses and streamlined workflows. AI-based applications continue to expand, automating tasks to improve efficiency and consistency, marking a promising shift toward a more efficient, patient-centered healthcare future (see https://sharedimaging.com/2024trends/, accessed on 26 August 2024).

Another trending topic is the search for solutions that utilize AI algorithms capable of enhancing image sensing by combining image sensors (e.g., depth sensors, thermal imaging, and multi-spectral imaging) with advanced computer vision algorithms. In addition, the integration of the Internet of Things (IoT) into imaging systems will further transform in-device connectivity and data exchange through the development of AI and IoT-based software [2]. This is manifested in this Special Issue within the provided systematic review [3].

Another impact of AI on imaging is generative AI, which uses generative models to create or manipulate images [4]. Modeling techniques such as generative adversarial networks and variational autoencoders create realistic-looking situations with applications in computer graphics, video games, and virtual reality environments. Virtual reality, for example, transforms complex image data into immersive and interactive experiences [5]. Users interact with data in three-dimensional space, facilitating better understanding, analysis, and decision-making.

As guest editor of this Special Issue and Section Editor-in-Chief of the collection “AI in Imaging” (https://www.mdpi.com/journal/jimaging/sections/AI_Imaging, accessed on 26 August 2024) with 53 papers published so far, I believe in the growing importance of AI in imaging in fields as diverse as computer science, engineering, biology, psychology, medicine, and neuroscience. I also believe that AI in imaging has not yet reached its full potential. I foresee a tremendous growth of solutions that leverage the AI/imaging binomial in the near future.
